# Pembrolizumab alone or in combination with chemotherapy as first-line therapy for patients with advanced gastric or gastroesophageal junction adenocarcinoma: results from the phase II nonrandomized KEYNOTE-059 study

**DOI:** 10.1007/s10120-018-00909-5

**Published:** 2019-03-25

**Authors:** Yung-Jue Bang, Yoon-Koo Kang, Daniel V. Catenacci, Kei Muro, Charles S. Fuchs, Ravit Geva, Hiroki Hara, Talia Golan, Marcelo Garrido, Shadia I. Jalal, Christophe Borg, Toshihiko Doi, Harry H. Yoon, Mary J. Savage, Jiangdian Wang, Rita P. Dalal, Sukrut Shah, Zev A. Wainberg, Hyun Cheol Chung

**Affiliations:** 1 Department of Internal Medicine, Seoul National University College of Medicine, 101, Daehak-ro, Jongno-gu, Seoul, 03080 Republic of Korea; 2 Division of Oncology, Department of Internal Medicine, Asan Medical Center, University of Ulsan, Seoul, Republic of Korea; 3 Section of Hematology/Oncology, Department of Medicine, University of Chicago Medicine, Chicago, IL USA; 4 Department of Clinical Oncology, Aichi Cancer Center Hospital, Nagoya, Aichi Japan; 5 Department of Medical Oncology, Yale Cancer Center, New Haven, CT USA; 6 Department of Oncology, Tel-Aviv Sourasky Medical Center, Tel Aviv University, Tel Aviv, Israel; 7 Department of Gastroenterology, Saitama Cancer Center, Saitama, Japan; 8 Department of Oncology, The Oncology Institute at the Chaim Sheba Medical Center, Sackler Faculty of Medicine, Tel Aviv University, Tel Aviv, Israel; 9 Hematology and Medical Oncology, Pontificia Universidad Católica de Chile, Santiago, Chile; 10 Department of Internal Medicine, Indiana University School of Medicine, Indianapolis, IN USA; 11 Department of Medical Oncology, University Hospital of Besançon, Besancon, France; 12 Department of Gastrointestinal Oncology, National Cancer Center Hospital East, Chiba, Kashiwa Japan; 13 Department of Medical Oncology, Mayo Clinic, Rochester, MN USA; 14 Medical Oncology, Merck & Co., Inc., Kenilworth, NJ USA; 15 Division of Hematology Oncology, David Geffen School of Medicine at UCLA, Los Angeles, CA USA; 16 Division of Medical Oncology, Yonsei Cancer Center, Yonsei University College of Medicine, Seoul, Republic of Korea

**Keywords:** Pembrolizumab, Cisplatin, 5-Fluorouracil, Capecitabine, Gastric cancer

## Abstract

**Background:**

The multicohort, phase II, nonrandomized KEYNOTE-059 study evaluated pembrolizumab ± chemotherapy in advanced gastric/gastroesophageal junction cancer. Results from cohorts 2 and 3, evaluating first-line therapy, are presented.

**Methods:**

Patients ≥ 18 years old had previously untreated recurrent or metastatic gastric/gastroesophageal junction adenocarcinoma. Cohort 3 (monotherapy) had programmed death receptor 1 combined positive score ≥ 1. Cohort 2 (combination therapy) received pembrolizumab 200 mg on day 1, cisplatin 80 mg/m^2^ on day 1 (up to 6 cycles), and 5-fluorouracil 800 mg/m^2^ on days 1–5 of each 3-week cycle (or capecitabine 1000 mg/m^2^ twice daily in Japan). Primary end points were safety (combination therapy) and objective response rate per Response Evaluation Criteria in Solid Tumors version 1.1 by central review, and safety (monotherapy).

**Results:**

In the combination therapy and monotherapy cohorts, 25 and 31 patients were enrolled; median follow-up was 13.8 months (range 1.8–24.1) and 17.5 months (range 1.7–20.7), respectively. In the combination therapy cohort, grade 3/4 treatment-related adverse events occurred in 19 patients (76.0%); none were fatal. In the monotherapy cohort, grade 3–5 treatment-related adverse events occurred in seven patients (22.6%); one death was attributed to a treatment-related adverse event (pneumonitis). The objective response rate was 60.0% [95% confidence interval (CI), 38.7–78.9] (combination therapy) and 25.8% (95% CI 11.9–44.6) (monotherapy).

**Conclusions:**

Pembrolizumab demonstrated antitumor activity and was well tolerated as monotherapy and in combination with chemotherapy in patients with previously untreated advanced gastric/gastroesophageal junction adenocarcinoma.

**Clinical Trial:**

**ClinicalTrials.gov** NCT02335411

**Electronic supplementary material:**

The online version of this article (10.1007/s10120-018-00909-5) contains supplementary material, which is available to authorized users.

## Introduction

Gastric cancer is the fifth most common malignancy worldwide. In 2012, it led to 723,000 deaths [1]. Most cases are advanced at diagnosis, and prognosis is poor [2, 3]. Chemotherapy containing fluoropyrimidine plus a platinum agent is standard first-line treatment for advanced gastric or gastroesophageal junction (G/GEJ) adenocarcinoma not expressing human epidermal growth factor receptor 2 (HER2) [4–6]. In previously untreated gastric cancer, this regimen demonstrated objective response rates (ORRs) of 25–48% and median overall survival (OS) of 8–11.2 months [7–12].

Tumor cells frequently use the programmed death receptor 1 (PD-1) pathway to evade immune surveillance [13, 14]. Binding of PD-1 to its ligands, PD-L1 and PD-L2, inhibits effector T-cell function, resulting in suppression of antitumor immune response [13, 14]. PD-L1 is frequently upregulated in gastric cancer [15–19]. Pembrolizumab is a selective, humanized, immunoglobulin G4κ monoclonal antibody that blocks interactions between PD-1 and its ligands [20]. The phase II KEYNOTE-059 clinical trial, a multicohort study designed to evaluate pembrolizumab alone or in combination with chemotherapy in patients with recurrent or metastatic G/GEJ adenocarcinoma, included a cohort of patients whose disease had progressed while they previously received at least two systemic therapies for advanced disease (cohort 1; pembrolizumab monotherapy) and two cohorts of patients who had not previously received systemic therapy for advanced disease. Data from cohort 1 have already been published and demonstrate durable responses with pembrolizumab monotherapy, with higher ORR and longer response durations in patients with PD-L1-positive tumors [combined positive score (CPS) ≥ 1] than with PD-L1-negative tumors [21]. Based on these results, the US Food and Drug Administration approved pembrolizumab for the treatment of patients with recurrent or metastatic G/GEJ adenocarcinoma whose tumors express PD-L1 and whose disease has progressed on or after ≥ 2 prior lines of therapy [20]. In the phase III, KEYNOTE-061 study, pembrolizumab did not significantly improve OS, versus paclitaxel, as second-line therapy in patients with advanced G/GEJ cancer and PD-L1 CPS ≥ 1 [22].

Chemotherapeutic agents such as cisplatin and 5-fluorouracil enhance immunogenicity of cancer cells and increase susceptibility to immune-mediated cytotoxicity [23–25]. Combining pembrolizumab with chemotherapy has demonstrated efficacy and manageable safety in other cancers [26, 27]. Cohorts 2 and 3 of the KEYNOTE-059 study were designed to further explore pembrolizumab in combination therapy and as monotherapy in the PD-L1-positive population. Herein, we present results from cohorts 2 and 3 of the KEYNOTE-059 study, which evaluated first-line therapy with pembrolizumab plus chemotherapy (cohort 2) or alone (cohort 3) in advanced G/GEJ cancer.

## Materials and methods

KEYNOTE-059 is a multicenter, open-label, nonrandomized, 3-cohort, phase II study. Results for cohorts 2 and 3 are presented; results for cohort 1 were published separately [21]. The study was conducted in accordance with Good Clinical Practice guidelines and the Declaration of Helsinki. The protocol was approved by institutional review boards and independent ethics committees for each institution. All patients provided written, informed consent. This trial was registered with ClinicalTrials.gov, number NCT02335411.

### Patients

Eligibility criteria for both cohorts were age ≥ 18 years, histologically or cytologically confirmed recurrent or metastatic G/GEJ adenocarcinoma, no prior systemic therapy for advanced disease, measurable disease per Response Evaluation Criteria in Solid Tumors version 1.1 (RECIST v1.1) by central review, HER2/neu-negative status, Eastern Cooperative Oncology Group performance status 0/1, life expectancy ≥ 3 months, and adequate organ function.

For cohort 2, patients had to provide new or archival tumor tissue for PD-L1 analysis. For cohort 3, patients had to have PD-L1-positive tumors as per the PD-L1 IHC 22C3 pharmDx assay (Agilent Technologies, Carpinteria, CA, USA). PD-L1 positivity was defined as CPS ≥ 1, where CPS is the number of PD-L1-positive cells (tumor cells, lymphocytes, and macrophages) divided by the total number of tumor cells multiplied by 100 [21]. Exclusion criteria are presented in Online Resource Table S1. We assessed DNA mismatch repair across 5 mononucleotide repeat markers (NR21, NR24, BAT25, BAT26, MONO27) using DNA extracted from formalin-fixed, paraffin-embedded tumor samples and blood (normal control) using the MSI Analysis System, version 1.2 (Promega). In microsatellite instability-high tumors, 2 or more markers were changed, compared with normal controls.

### Study treatment

In cohort 2, patients received pembrolizumab 200 mg by intravenous infusion over 30 min on day 1, cisplatin 80 mg/m^2^ on day 1 (for up to 6 cycles), and 5-fluorouracil 800 mg/m^2^ by continuous infusion on days 1–5 of each 21-day cycle. In Japan only, patients received capecitabine 1000 mg/m^2^ twice daily orally on days 1–14 of each 21-day cycle instead of 5-fluorouracil. Up to two dose reductions were allowed per chemotherapy agent (Online Resource Table S2). In cohort 3, patients received pembrolizumab monotherapy 200 mg by intravenous infusion over 30 min on day 1 of each 21-day cycle.

Pembrolizumab dose reduction was not permitted. Treatment was continued for up to 35 cycles (~ 2 years) or until confirmed disease progression, unacceptable toxicity, complete response, or patient or investigator decision to withdraw. Clinically stable patients with radiographic progressive disease (PD) could continue treatment at the investigator’s discretion until confirmed PD. Patients in cohort 2 who discontinued chemotherapy could continue pembrolizumab if they had not met criteria for discontinuation.

### Outcomes and assessments

For cohort 2, the primary end points were safety and tolerability. Secondary end points included ORR (percentage of patients who experienced complete or partial response) per RECIST v1.1 by central imaging review and evaluation of the relationship between PD-L1 expression and efficacy. For cohort 3, primary end points were ORR and safety and tolerability. Secondary end points included disease control rate (DCR; percentage of patients who experienced complete or partial response or stable disease for ≥ 6 months), duration of response, progression-free survival (PFS; time from study treatment initiation to first documented instance of PD or death) per RECIST v1.1 by central imaging review, and OS (time from initiation of study treatment to death from any cause).

Tumors were assessed by computed tomography or magnetic resonance imaging at 9 weeks after study treatment initiation, every 6 weeks for year 1, and every 9 weeks thereafter (or more often if clinically indicated). Response was confirmed by repeat radiographic assessment ≥ 4 weeks after first documentation. Patients were followed up every 12 weeks for survival status until death, withdrawal of consent, or study end.

Patients were monitored for adverse events (AEs) until 30 days after the last dose of treatment (90 days for serious AEs and events of clinical interest). AEs were graded using National Cancer Institute Common Terminology Criteria for Adverse Events, version 4.0; investigators determined treatment attribution.

### Statistical analysis

For cohort 2, the sample size was based on clinical experience. A sample size of ~ 18 patients was planned to provide the reasonable two-sided 95% confidence interval (CI) of AE incidence (Online Resource Table S3). For cohort 3, a sample size of ~ 25 patients with PD-L1 CPS ≥ 1 was estimated using Bayesian sample size methods assuming a uniform prior for the response rate and a true ORR of < 40% (ORR based on standard of care). Safety and efficacy were analyzed in all patients who received ≥ 1 dose of study treatment. Safety was analyzed using descriptive statistics. Analyses were conducted using SAS statistical software version 9.3 (SAS Institute, Inc). ORR and DCR with 95% CI were calculated using the exact method based on binomial distribution (Clopper–Pearson method [28]). Patients with missing data were considered nonresponders. Time-to-event end points were estimated using the Kaplan–Meier method, with censoring at the last date of assessment for patients with missing data.

## Results

The study was conducted at 57 sites in 17 countries. For cohort 2, 25 patients were enrolled at 10 sites in the Republic of Korea (South Korea), Japan, Israel, the United States, and France between March 2, 2015, and December 14, 2015. For cohort 3, 31 patients were enrolled at 18 sites in Canada, Chile, Israel, Japan, South Korea, and the United States between July 23, 2015, and December 2, 2015. Patient characteristics are presented in Table 1.

**Table 1:** Patient characteristics

Characteristic	Cohort 2 *N* = 25	Cohort 3 *N* = 31
Male	16 (64.0)	19 (61.3)
Age, median (range) (years)	64 (21–82)	62 (32–75)
Race		
Asian	17 (68.0)	15 (48.4)
White	8 (32.0)	16 (51.6)
Region		
United States	3 (12.0)	12 (38.7)
East Asia	16 (64.0)	13 (41.9)
Rest of world	6 (24.0)	6 (19.4)
ECOG performance status		
0	15 (60.0)	14 (45.2)
1	10 (40.0)	17 (54.8)
Metastatic stage		
M0	1 (4.0)	5 (16.1)
M1	24 (96.0)	26 (83.9)
Prior surgery for gastric cancer	5 (20.0)	12 (38.7)
Histology, WHO classification		
Tubular adenocarcinoma	22 (88.0)	25 (80.6)
Signet ring cell carcinoma	2 (8.0)	3 (9.7)
Mixed carcinoma	1 (4.0)	2 (6.5)
Other poorly cohesive carcinoma	0	1 (3.2)
PD-L1 expression		
CPS ≥ 1	16 (64.0)	31 (100.0)
CPS < 1	8 (32.0)	0
Unknown	1 (4.0)	0

Unless otherwise indicated, all data are *n* (%)

*CPS* combined positive score, *ECOG* Eastern Cooperative Oncology Group, *PD-L1* programmed death ligand 1, *WHO* World Health Organization

At data cutoff (April 21, 2017), median follow-up was 13.8 months (range 1.8–24.1) in cohort 2 and 17.5 months (range 1.7–20.7) in cohort 3. In cohort 2, 22 patients (88.0%) had discontinued study treatment and three (12.0%) remained on treatment. In cohort 3, 24 patients (77.4%) had discontinued study treatment, whereas seven patients (22.6%) remained on treatment. In both cohorts, the most common reason for treatment discontinuation was PD (Online Resource Figure S1, CONSORT).

### Treatment exposure and safety

#### Cohort 2

Median duration of pembrolizumab exposure was 7.1 months (range 0.8–23.8), and median number of administrations was 10 (range 2–35). All patients experienced treatment-related AEs (TRAEs) (Table 2). Grade 3 TRAEs occurred in 15 patients (60.0%). One TRAE was grade 4 (decreased neutrophil count, 16.0%). No TRAEs were fatal. AEs and serious AEs related to chemotherapy and pembrolizumab are presented in Online Resource Tables S4 and S5. TRAEs leading to treatment interruption are described in Online Resource Table S6. Three patients (12.0%) discontinued treatment because of chemotherapy-related AEs: grade 3 stomatitis, grade 2 hypoacusis, and grade 1 increased creatinine level. No patient discontinued treatment because of pembrolizumab-related AEs. Immune-mediated AEs (regardless of attribution) occurred in 12 patients (48.0%) and were grade 3 in four patients (16.0%) (Online Resource Table S7). No grade 4/5 immune-mediated AEs occurred. Five of 12 patients who experienced an immune-mediated AE (41.7%) received systemic corticosteroids.

**Table 2:** Treatment-related AEs of any grade occurring in ≥ 10% of patients and grade 3 treatment-related AEs occurring in ≥ 1 patients in cohort 2

Treatment-related AEs^a^*n* (%)	Cohort 2 *N* = 25	
	Any grade	Grade 3
Any	25 (100.0)	15 (60.0)
Hematologic		
Decreased neutrophil count	21 (84.0)	12 (48.0)
Anemia	5 (20.0)	2 (8.0)
Decreased platelet count	8 (32.0)	2 (8.0)
Decreased WBC count	4 (16.0)	1 (4.0)
Febrile neutropenia	1 (4.0)	1 (4.0)
Nonhematologic		
Nausea	13 (52.0)	1 (4.0)
Stomatitis^b^	13 (52.0)	5 (20.0)
Decreased appetite	11 (44.0)	2 (8.0)
Fatigue	8 (32.0)	2 (8.0)
Diarrhea	8 (32.0)	0
Dysgeusia	7 (28.0)	0
Constipation	6 (24.0)	0
Vomiting	6 (24.0)	0
Hiccups	5 (20.0)	0
Malaise	5 (20.0)	0
Palmar–plantar erythrodysesthesia syndrome	4 (16.0)	2 (8.0)
Alopecia	4 (16.0)	0
Peripheral sensory neuropathy	4 (16.0)	0
Hyperthyroidism	4 (16.0)	0
Maculopapular rash or rash	4 (16.0)	2 (8.0)
Pyrexia	3 (12.0)	1 (4.0)
Decreased weight	3 (12.0)	1 (4.0)
Increased blood creatinine level	3 (12.0)	0
Mucosal inflammation^c^	3 (12.0)	0
Peripheral neuropathy	3 (12.0)	0
Increased weight	3 (12.0)	0
Hypophosphatemia	1 (4.0)	1 (4.0)
Polymyalgia rheumatica	1 (4.0)	1 (4.0)

*AE* adverse event, *WBC* white blood cell

^a^Attribution of AEs to study treatment was determined by the investigator

^b^Stomatitis refers to inflammation of the mouth and lips

^c^Mucosal inflammation refers to inflammation of the mucous membranes

#### Cohort 3

Median duration of exposure to pembrolizumab was 2.8 months (range 0.7–20.3), and median number of administrations was five (range 2–30). Overall, TRAEs occurred in 24 patients (77.4%) (Table 3). Grade 3–5 TRAEs occurred in seven patients (22.6%). One patient died because of pneumonitis 74 days after the last pembrolizumab dose. Serious TRAEs occurred in six patients (19.4%). TRAEs resulted in treatment interruption in eight patients (25.8%) (Online Resource Table S8). No patient discontinued because of a TRAE. Immune-mediated AEs occurred in 10 patients (32.3%) and were grade 3–5 in three patients (9.7%) (Online Resource Table S9). Four of ten patients who experienced an immune-mediated AE (40.0%) received systemic corticosteroids.

**Table 3:** Treatment-related AE of any grade occurring in ≥ 10% of patients and grade 3 treatment-related AEs occurring in ≥ 1 patient in cohort 3

Treatment-related AEs^a^***n*(%)**	Cohort 3 ***N*** = **31**	
	Any grade	Grade 3
Any	24 (77.4)	6 (19.4)
Fatigue	8 (25.8)	0
Pruritus	7 (22.6)	0
Pneumonitis	4 (12.9)	0
Bile duct obstruction	1 (3.2)	1 (3.2)
Colitis	1 (3.2)	1 (3.2)
Dehydration	1 (3.2)	1 (3.2)
Diffuse uveal melanocytic proliferation	1 (3.2)	1 (3.2)
Hyponatremia	1 (3.2)	1 (3.2)
Neutropenia	1 (3.2)	1 (3.2)
Rash	1 (3.2)	1 (3.2)

*AE* adverse event

^a^Attribution of AEs to study treatment was determined by the investigator

### Antitumor activity

#### Cohort 2

Confirmed ORR was 60.0% (95% CI 39.0–79.0), with complete response in one patient (4.0%) (Table 4). Median time to response was 2.1 months (range 1.9–3.7) and median response duration was 4.6 months (range 2.6–20.3+). At data cutoff, response was ongoing in two of 15 responders (13.3%), with response durations of 20.0 months and 20.3 months, respectively. No patient in cohort 2 had microsatellite instability-high tumors.

**Table 4:** Antitumor activity^a^ assessed by central review per RECIST v1.1 and duration of response

Category	Cohort 2 *N* = 25		Cohort 3 *N* = 31	
	*n*	% (95% CI^b^)	*n*	% (95% CI^b^)
Objective response rate^c^	15	60.0 (38.7–78.9)	8	25.8 (11.9–44.6)
Disease control rate^d^	20	80.0 (59.3–93.2)	11	35.5 (19.2–54.6)
Best overall response				
Complete response	1	4.0 (0.1–20.4)	2	6.5 (0.8–21.4)
Partial response	14	56.0 (34.9–75.6)	6	19.4 (7.5–37.5)
Stable disease	8	32.0 (14.9–53.5)	9	29.0 (14.2–48.0)
Progressive disease	1	4.0 (0.1–20.4)	12	38.7 (21.8–57.8)
Nonevaluable/no assessment	1	4.0 (0.1–20.4)	2	6.5 (0.8–21.4)
Median (range) time to response (months)	2.1 (1.9–3.7)		2.1 (1.5–6.2)	
Median (range) duration of response (months)	4.6 (2.6–20.3+)		9.6 (2.1–17.8+)	

The + indicates that there was no progressive disease at last disease assessment

*CI* confidence interval, *RECIST* Response Evaluation Criteria in Solid Tumors

^a^Confirmed by repeat radiographic assessment ≥ 4 weeks after first documentation of response

^b^Based on binomial exact CI method

^c^Complete response + partial response

^d^Complete response + partial response + stable disease maintained for ≥ 6 months

**Fig. 1:**
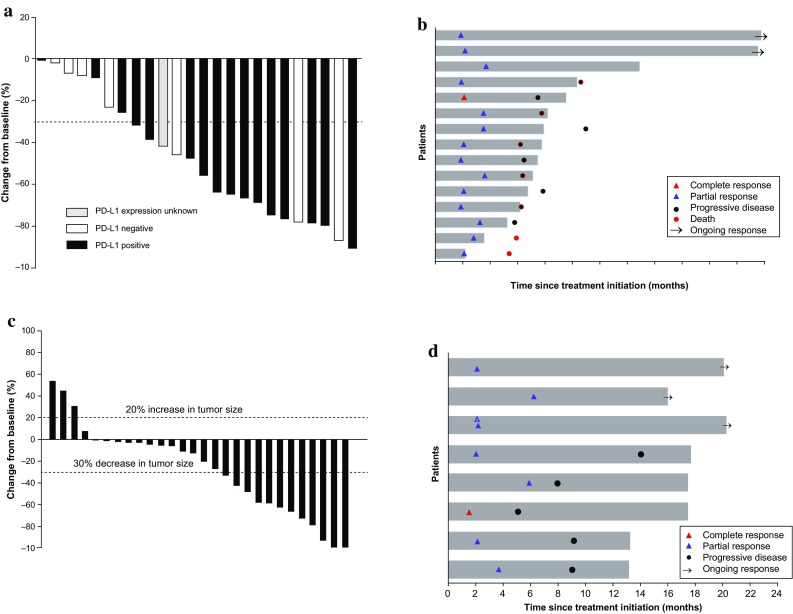
Antitumor activity. **a** Best change from baseline in the sum of longest target lesion diameters per patient by PD-L1 expression in cohort 2 (*n* = 24)^a^. **b** Duration of exposure and best response in confirmed responders in cohort 2 (*n* = 15)^b^. **c** Maximum percentage change from baseline in the sum of longest diameter of target lesions per patient (*n* = 28)^a^. **d** Duration of exposure and best response in confirmed responders (*n* = 8)^b^. ^a^Patients with measurable disease per RECIST v1.1 by central review at baseline who had ≥ 1 evaluable postbaseline assessment. ^b^Patients with measurable disease per RECIST v1.1 by central review at baseline who had ≥ 1 postbaseline assessment and had confirmed response. Bar length indicates time to last dose of study drug. Time to first confirmed response is shown. PD-L1, programmed death ligand 1; RECIST v1.1, Response Evaluation Criteria in Solid Tumors version 1.1

Reductions in target lesion size from baseline were observed in all 24 patients (100%) who had measurable disease at baseline and ≥ 1 evaluable postbaseline assessment (Fig. 1a). Responders generally experienced reduction in tumor burden within a few months (Fig. 1b; Online Resource Figure S2).

In cohort 2, 16 patients had PD-L1 CPS ≥ 1 and eight had PD-L1 CPS < 1. Among the 16 patients with PD-L1 CPS ≥ 1, ORR was 68.8% (95% CI 41.3–89.0%; Online Resource Table S10), median time to response was 2.1 months (range 1.9–3.5), and median duration of response was 4.6 months (range 3.2–18.1+). Among the eight patients with PD-L1 CPS < 1, ORR was 37.5% (95% CI 8.5–75.5%), median time to response was 3.6 months (range 2.1–3.7), and median duration of response was 5.4 months (range 2.8–10.1+).

Among 19 patients receiving pembrolizumab, cisplatin, and 5-fluorouracil, ORR was 57.9% (96% CI 33.5–79.7%). Among six Japanese patients receiving pembrolizumab, cisplatin, and capecitabine, ORR was 66.7% (95% CI 22.3–95.7%) (Online Resource Table S11).

#### Cohort 3

Confirmed ORR was 25.8% (95% CI 11.9–44.6%), with complete response in two patients (6.5%) (Table 3). Median time to response was 2.1 months (range 1.5–6.2) and median response duration was 9.6 months (range 2.1–17.8+) (Table 3). Response was ongoing at data cutoff in three of eight responders (37.5%), with response durations of 17.4, 13.7, and 17.8 months. One patient in cohort 3 had microsatellite instability-high tumors and experienced a partial response.

Of 28 patients with measurable disease at baseline who had ≥ 1 evaluable postbaseline assessment, 24 (85.7%) experienced reduction in target lesion size from baseline (Fig. 1c). Decrease in tumor burden was generally maintained over several assessments (Fig. 1d; Online Resource Figure S2).

### Survival outcomes

#### Cohort 2

**Fig. 2:**
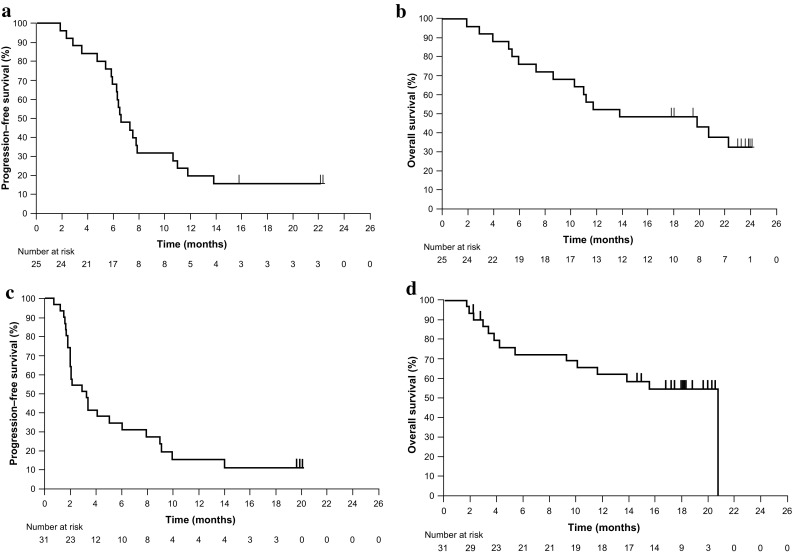
Kaplan–Meier estimates of **a** progression-free survival and **b** overall survival in cohort 2, and **c** progression-free survival and **d** overall survival in cohort 3

Median PFS was 6.6 months (95% CI 5.9–10.6), and estimated PFS rate at 6 months was 68.0% (Fig. 2a). Median OS was 13.8 months (95% CI 8.6—not estimable), and estimated OS rates were 52% and 48% at 12 and 18 months, respectively (Fig. 2b). In patients with PD-L1 CPS ≥ 1 and < 1, median OS was 11.1 months (95% CI 5.4–22.3) and 19.8 months (95% CI 1.8—not estimable), respectively.

#### Cohort 3

Median PFS was 3.3 months (95% CI 2.0–6.0 months), and estimated PFS rate at 6 months was 34.9% (Fig. 2c). Median OS was 20.7 months (95% CI 9.2–20.7), and estimated OS rates were 63% and 55% at 12 and 18 months, respectively (Fig. 2d).

## Discussion

Cohort 1 of the KEYNOTE-059 study confirmed that pembrolizumab monotherapy provided durable responses with manageable safety in patients with advanced G/GEJ adenocarcinoma that had progressed following second-line treatment [21]. Although patient numbers in the current analyses were small, results from cohorts 2 and 3 of the KEYNOTE-059 study demonstrated manageable safety and antitumor activity for pembrolizumab as monotherapy and as combination therapy with cisplatin plus 5-fluorouracil or capecitabine in previously untreated advanced G/GEJ adenocarcinoma. Although it is difficult to draw definitive conclusions regarding the clinical impact of pembrolizumab based solely on these data, the results, along with those from cohort 1 of the study [21], support the growing body of evidence for the potential use of pembrolizumab in patients with G/GEJ adenocarcinoma. Additional large-scale clinical trials are needed to confirm efficacy and safety in these patients.

In cohort 2, the incidence and severity of TRAEs with pembrolizumab plus cisplatin plus 5-fluorouracil or capecitabine were generally consistent with those of known toxic effects of cisplatin plus fluoropyrimidine [7, 9, 10, 12]. The incidence of grades 3 and 4 chemotherapy-related neutropenia (48% and 16%, respectively) in this cohort was somewhat higher than expected; previous studies of first-line chemotherapy in patients with advanced gastric cancer have reported grade ≥ 3 neutropenia occurring at rates of 20–44% [29–31], although these data were collected from larger study populations that may not be directly comparable. It is notable that neutropenia levels were not elevated in either cohort 3 or cohort 1 [21], but, with a population of just 25 patients, it is difficult to draw definitive conclusions as to the cause of the slightly greater incidence in cohort 2. The incidence of pembrolizumab-related stomatitis (12% overall; 8% grade 3) also initially appeared to be higher than expected, but this was likely an artifact given the small number of patients involved (3 of 25). Although a similar incidence of pembrolizumab-related stomatitis (2 of 23; 9%) was reported in a recently published study of patients with advanced colorectal carcinoma [32], the risk associated with pembrolizumab appears to be small compared with the number of patients in whom chemotherapy-related stomatitis develops (52% in the current study; 16% grade ≥ 3). Immune-mediated AEs were similar to those reported with pembrolizumab plus platinum-based doublets in non-small cell lung cancer [26], suggesting a degree of constancy across nonhematologic malignancies.

In cohort 3, the safety profile of pembrolizumab monotherapy was consistent with that previously reported [21, 33]. No new safety signals were identified. Relative to standard chemotherapy, pembrolizumab had lower rates of grade 3/4 AEs and discontinuation because of AEs, suggesting potential for a greater therapeutic index with pembrolizumab than with available therapies [7–12] and the possibility of combining with other therapies.

Although study designs and eligibility criteria differ across trials, the ORR of 60.0% (95% CI 39.0–79.0%) with pembrolizumab plus cisplatin and 5-fluorouracil or capecitabine in cohort 2 is encouraging relative to response rates observed with cisplatin and fluoropyrimidine doublets (25–48%) [7–12]. The ORR of 25.8% (95% CI 11.9–44.6%) with pembrolizumab monotherapy in patients with PD-L1 CPS ≥ 1 was also within the range expected with chemotherapy (25–48%) [7–12]. Additionally, median OS in both cohorts compares favorably with the OS with chemotherapy (8–11.2 months), as does 1-year OS [7–12].

Sixty-four percent of patients in cohort 2 were PD-L1 positive (CPS ≥ 1), compared with an overall prevalence of PD-L1 positivity in advanced gastric cancer of 30–65%, although different methods, antibodies, and cutoff values were used across studies [15–19]. Analysis of patients with PD-L1 CPS ≥ 1 in cohort 2 suggests that PD-L1 expression enhances response to pembrolizumab plus chemotherapy; however, larger prospective clinical trials are necessary to substantiate these results.

One patient in cohort 3 who had microsatellite instability-high tumors experienced a partial response. Although this partial response contributed to the ORR in cohort 3, it does not account for the ORR in its entirety. This finding is consistent with that reported for pembrolizumab monotherapy in the cohort of patients who have received ≥ 2 prior therapies for G/GEJ cancer in this KEYNOTE-059 study [21].

Overall, pembrolizumab plus cisplatin and 5-fluorouracil or capecitabine demonstrated manageable safety and promising antitumor activity as first-line therapy in advanced G/GEJ adenocarcinoma, regardless of PD-L1 expression. Pembrolizumab monotherapy also demonstrated encouraging antitumor activity and acceptable safety in patients with PD-L1 CPS ≥ 1. Results support continued evaluation of pembrolizumab in clinical trials.

## Electronic supplementary material

Below is the link to the electronic supplementary material.

Supplementary material 1 (DOCX 1069 KB)
