# Supplementary material for: Pembrolizumab alone or in combination with chemotherapy as first-line therapy for patients with advanced gastric or gastroesophageal junction adenocarcinoma: results from the phase II nonrandomized KEYNOTE-059 study
Source: Gastric Cancer. 2019 Mar 25;22(4):828–37. doi: 10.1007/s10120-018-00909-5 (PMC6570680; doi:10.1007/s10120-018-00909-5)

Journal: *Gastric Cancer*

**Pembrolizumab alone or in combination with chemotherapy as first-line therapy for patients with advanced gastric or gastroesophageal junction adenocarcinoma: results from the phase II nonrandomized KEYNOTE-059 study**

Yung-Jue Bang^1*†^, Yoon-Koo Kang^2*†^, Daniel V. Catenacci^3^, Kei Muro^4^, Charles S. Fuchs^5^, Ravit Geva^6^, Hiroki Hara^7^, Talia Golan^8^, Marcelo Garrido^9^, Shadia I. Jalal^10^, Christophe Borg^11^, Toshihiko Doi^12^, Harry H. Yoon^13^, Mary J. Savage^14^, Jiangdian Wang^14^, Rita P. Dalal^14‡^, Sukrut Shah^14^, Zev A. Wainberg^15^, Hyun Cheol Chung^16†^

^1^Department of Internal Medicine, Seoul National University College of Medicine, Seoul, Republic of Korea; ^2^Division of Oncology, Department of Internal Medicine, Asan Medical Center, University of Ulsan, Seoul, Republic of Korea; ^3^Department of Medicine, Section of Hematology/Oncology, University of Chicago Medicine, Chicago, IL, USA; ^4^Department of Clinical Oncology, Aichi Cancer Center Hospital, Nagoya, Aichi, Japan; ^5^Department of Medical Oncology, Yale Cancer Center, New Haven, CT; ^6^Department of Oncology, Tel-Aviv Sourasky Medical Center, Tel Aviv University, Tel Aviv, Israel; ^7^Department of Gastroenterology, Saitama Cancer Center, Saitama, Japan; ^8^Department of Oncology, The Oncology Institute at the Chaim Sheba Medical Center, Sackler Faculty of Medicine, Tel Aviv University, Tel Aviv, Israel; ^9^Hematology and Medical Oncology, Pontificia Universidad Católica de Chile, Santiago, Chile; ^10^Department of Internal Medicine, Indiana University School of Medicine, Indianapolis, IN, USA; ^11^Medical Oncology, University Hospital of Besançon, Besancon, France; ^12^Department of Gastrointestinal Oncology, National Cancer Center Hospital East, Chiba, Kashiwa, Japan; ^13^Department of Medical Oncology, Mayo Clinic, Rochester, MN, USA; ^14^Medical Oncology, Merck & Co., Inc., Kenilworth, NJ, USA; ^15^Division of Hematology Oncology, David Geffen School of Medicine at UCLA, Los Angeles, CA, USA; ^16^Division of Medical Oncology, Yonsei Cancer Center, Yonsei University College of Medicine, Seoul, Republic of Korea

^†^Contributed equally

^‡^At the time of the study

**Correspondence to:** Dr Yung-Jue Bang, Department of Internal Medicine, Seoul National University College of Medicine, 101, Daehak-ro, Jongno-gu, Seoul, 03080, Republic of Korea.

Telephone: +82-22-072-2362

Fax: +82 02-3675-8333

Email: bangyj@snu.ac.kr

**Table S1** Exclusion criteria

| Active autoimmune disease necessitating systemic therapy within the preceding 2 years |
| --- |
| Immunodeficiency |
| Receiving systemic corticosteroids or other immunosuppressive therapy within 7 days before study start |
| Active central nervous system metastases and/or carcinomatous meningitis |
| Known history of active, noninfectious pneumonitis |
| Active infection necessitating systemic therapy |
| Prior treatment with agents targeting PD-1, PD-L1, or PD-L2 |

# PD-1, programmed death 1; PD-L1, programmed death ligand 1; PD-L2, programmed death ligand 2.

# **Table S2** Dose modifications for study medications in cohort 2

| **Medication** | **Starting dose** | **Dose level 1** | **Dose level 2** | **Dose level 3** |
| --- | --- | --- | --- | --- |
| Cisplatin | 80 mg/m^2^ | 60 mg/m^2^ | 40 mg/m^2^ | Discontinue |
| 5-Fluorouracil | 800 mg/m^2^ | 600 mg/m^2^ | 400 mg/m^2^ | Discontinue |
| Capecitabine (used only in Japan) | 1000 mg/m^2^ BID | 750 mg/m^2^ BID | 500 mg/m^2^ BID | Discontinue |

BID, twice daily.

# **Table S3** Two-sided 95% CI of AE incidence rate with 18 patients (cohort 2)

| **AEs, *n*** | **AE incidence rate estimates (%)** | **95% CI of incidence rate (%)** |
| --- | --- | --- |
| 2 | 11.1 | 1.4–34.7 |
| 4 | 22.2 | 6.4–47.6 |
| 5 | 27.7 | 9.7–53.5 |
| 7 | 38.9 | 17.3–64.2 |
| 9 | 50.0 | 26.0–73.9 |

AE, adverse event; CI, confidence interval.

# **Table S4** Chemotherapy-related^a^ and pembrolizumab-related^a^ AEs occurring in ≥10% of patients in cohort 2

| **Treatment-related AEs, *n* (%)** | **Cohort 2 *N* = 25** | | |
| --- | --- | --- | --- |
|  | **Any grade** | **Grade 3** | **Grade 4** |
| **Chemotherapy related^a^** | | | |
| Neutropenia | 21 (84.0) | 12 (48.0) | 4 (16.0) |
| Nausea | 13 (52.0) | 1 (4.0) | 0 |
| Stomatitis | 13 (52.0) | 4 (16.0) | 0 |
| Decreased appetite | 11 (44.0) | 2 (8.0) | 0 |
| Diarrhea | 8 (32.0) | 0 | 0 |
| Fatigue | 7 (28.0) | 1 (4.0) | 0 |
| Vomiting | 6 (24.0) | 0 | 0 |
| Dysgeusia | 6 (24.0) | 0 | 0 |
| Hiccups | 5 (20.0) | 0 | 0 |
| Malaise | 5 (20.0) | 0 | 0 |
| Constipation | 5 (20.0) | 0 | 0 |
| Anemia | 5 (20.0) | 2 (8.0) | 0 |
| Decreased platelet count | 8 (32.0) | 2 (8.0) | 0 |
| Decreased white blood cell count | 4 (16.0) | 1 (4.0) | 0 |
| Peripheral sensory neuropathy | 4 (16.0) | 0 | 0 |
| Alopecia | 4 (16.0) | 0 | 0 |
| Palmar–plantar erythrodysesthesia syndrome | 4 (16.0) | 2 (8.0) | 0 |
| Mucosal inflammation | 3 (12.0) | 0 | 0 |
| Increased blood creatinine | 3 (12.0) | 0 | 0 |
| Peripheral neuropathy | 3 (12.0) | 0 | 0 |
| Decreased weight | 3 (12.0) | 1 (4.0) | 0 |
| Increased weight | 3 (12.0) | 0 | 0 |
| **Pembrolizumab related^a^** | | | |
| Hyperthyroidism | 4 (16.0) | 0 | 0 |
| Stomatitis | 3 (12.0) | 2 (8.0) | 0 |
| Dysgeusia | 3 (12.0) | 0 | 0 |
| Maculopapular rash | 3 (12.0) | 1 (4.0) | 0 |

AE, adverse event.
^a^Attribution of AEs to study treatment was determined by the investigator.

# **Table S5** Chemotherapy-related^a^ and pembrolizumab-related^a^ serious AEs in cohort 2

| **Serious AEs, *n* (%)** | **Cohort 2**  ***N* = 25** |
| --- | --- |
| **Chemotherapy related^a^** | |
| Any | 4 (12.0) |
| Stomatitis | 3 (12.0) |
| Decreased appetite | 2 (8.0) |
| Seizure | 1 (4.0) |
| Palmar–plantar erythrodysesthesia syndrome | 1 (4.0) |
| **Pembrolizumab related^a^** | |
| Any | 3 (12.0) |
| Stomatitis | 2 (8.0) |
| Maculopapular rash | 2 (8.0) |
| Pyrexia | 1 (4.0) |
| Seizure | 1 (4.0) |
| Palmar–plantar erythrodysesthesia syndrome | 1 (4.0) |

AE, adverse event.
^a^Attribution of AEs to study treatment was determined by the investigator.

# **Table S6** Treatment interruptions owing to chemotherapy-related^a^ and pembrolizumab-related^a^ AEs in cohort 2

| **AEs leading to treatment interruption, *n* (%)** | **Cohort 2  *N* = 25** |
| --- | --- |
| **Chemotherapy-related^a^** | |
| Any | 21 (84.0) |
| Neutropenia | 15 (60.0) |
| Decreased appetite | 4 (16.0) |
| Stomatitis | 2 (8.0) |
| Fatigue | 2 (8.0) |
| Malaise | 1 (4.0) |
| Febrile neutropenia | 1 (4.0) |
| Anemia | 1 (4.0) |
| Cellulitis | 1 (4.0) |
| Infusion-related reaction | 1 (4.0) |
| Decreased platelet count | 1 (4.0) |
| Decreased weight | 1 (4.0) |
| Decreased white blood cell count | 1 (4.0) |
| **Pembrolizumab-related^a^** | |
| Any | 6 (24.0) |
| Neutropenia | 1 (4.0) |
| Stomatitis | 1 (4.0) |
| Colitis | 1 (4.0) |
| Increased aspartate aminotransferase level | 1 (4.0) |
| Myositis | 1 (4.0) |
| Polymyalgia rheumatica | 1 (4.0) |
| Scleroderma | 1 (4.0) |
| Rash | 1 (4.0) |
| Maculopapular rash | 1 (4.0) |

AE, adverse event.
^a^Attribution of AEs to study treatment was determined by the investigator.

**Table S7**  Immune-mediated AEs and infusion-related reactions, regardless of attribution to study treatment, in cohort 2

| **Immune-mediated AEs, *n* (%)** | **Cohort 2  *N* = 25** | |
| --- | --- | --- |
|  | **Any grade** | **Grade 3^a^** |
| Any | 12 (48.0) | 4 (16.0) |
| Hyperthyroidism | 4 (16.0) | 0 |
| Severe skin reactions^b^ | 3 (12.0) | 3 (12.0) |
| Hypothyroidism | 2 (8.0) | 0 |
| Colitis | 1 (4.0) | 0 |
| Infusion-related reaction | 1 (4.0) | 0 |
| Myositis | 1 (4.0) | 0 |
| Nephritis | 1 (4.0) | 1 (4.0) |
| Pneumonitis | 1 (4.0) | 0 |
| Thyroiditis | 1 (4.0) | 0 |

AE, adverse event.
^a^There were no grade 4/5 immune-mediated or infusion-related reactions.
^b^Includes rash, maculopapular rash, and palmar–plantar erythrodysesthesia.

# **Table S8** Treatment interruptions owing to treatment-related AEs^a^ in cohort 3

| **AEs leading to treatment interruption, *n* (%)** | **Cohort 3**  ***N* = 31** |
| --- | --- |
| Any | 8 (25.8) |
| Pneumonitis | 2 (6.5) |
| Neutropenia | 1 (3.2) |
| Adrenal insufficiency | 1 (3.2) |
| Diffuse uveal melanocytic proliferation | 1 (3.2) |
| Colitis | 1 (3.2) |
| Bile duct obstruction | 1 (3.2) |
| Epididymitis | 1 (3.2) |
| Rash | 1 (3.2) |

AE, adverse event.

^a^Attribution of AEs to study treatment was determined by the investigator.

# **Table S9** Immune-mediated AEs and infusion-related reactions, regardless of attribution to study treatment, in cohort 3

| **Immune-mediated AEs *n* (%)** | **Cohort 3 *N* = 31** | |
| --- | --- | --- |
|  | **Any grade** | **Grade 3-5** |
| Any | 10 (32.3) | 3 (9.7) |
| Pneumonitis | 4 (12.9) | 1 (3.2) |
| Hyperthyroidism | 2 (6.5) | 0 |
| Hypothyroidism | 2 (6.5) | 0 |
| Adrenal insufficiency | 1 (3.2) | 0 |
| Colitis | 1 (3.2) | 1 (3.2) |
| Infusion reactions | 1 (3.2) | 0 |
| Severe skin reactions^a^ | 1 (3.2) | 1 (3.2) |

AE, adverse event.

^a^Includes one event of grade 3 rash.

**Table S10**  Antitumor activity^a^ of pembrolizumab plus cisplatin plus 5-fluorouracil or capecitabine by PD-L1 expression in cohort 2

| Category | **PD-L1 CPS ≥1  *n* = 16** | | **PD-L1 CPS <1 *n* = 8** | |
| --- | --- | --- | --- | --- |
|  | ***n*** | **% (95% CI^b^)** | ***n*** | **% (95% CI^b^)** |
| Objective response rate^c^ | 11 | 68.8 (41.3–89.0) | 3 | 37.5 (8.5–75.5) |
| Disease control rate^d^ | 13 | 81.3 (54.4–96.0) | 6 | 75.0 (34.9–96.8) |
| Best overall response | | | | |
| Complete response | 0 | 0.0 (0.0–20.6) | 1 | 12.5 (0.3–52.7) |
| Partial response | 11 | 68.8 (41.3–89.0) | 2 | 25.0 (3.2–65.1) |
| Stable disease | 4 | 25.0 (7.3–52.4) | 4 | 50.0 (15.7–84.3) |
| Progressive disease | 1 | 6.3 (0.2–30.2) | 0 | 0.0 (0.0–36.9) |
| Nonevaluable | 0 | 0.0 (0.0–21.8) | 1 | 12.5 (0.3–52.7) |
| Median response duration (range) (months) | 4.6 (3.2 to 20.3+) | | 5.4 (2.8 to 12.0+) | |

CI, confidence interval; CPS, combined positive score; PD-L1, programmed death ligand 1.

+ indicates that there was no progressive disease at last disease assessment.

^a^Confirmed by repeat radiographic assessment ≥4 weeks after first documentation of response.

^b^Based on binomial exact CI method.

^c^Complete response + partial response.

^d^Complete response + partial response + stable disease maintained for ≥6 months.

**Table S11**  Antitumor activity^a^ of pembrolizumab plus cisplatin plus 5-fluorouracil versus pembrolizumab plus cisplatin plus capecitabine in cohort 2

| Category | **Pembrolizumab + cisplatin + 5-FU**  ***n* = 19** | | **Pembrolizumab + cisplatin + capecitabine**  ***n* = 6** | |
| --- | --- | --- | --- | --- |
|  | ***n*** | **% (95% CI^b^)** | ***n*** | **% (95% CI^b^)** |
| Objective response rate^c^ | 11 | 57.9 (33.5–79.7) | 4 | 66.7 (22.3–95.7) |
| Disease control rate^d^ | 15 | 78.9 (54.4–93.9) | 5 | 83.3 (35.9–99.6) |
| Best overall response | | | | |
| Complete response | 0 | 0.0 (0.0–17.6) | 1 | 16.7 (0.4–64.1) |
| Partial response | 11 | 57.9 (33.5–79.7) | 3 | 50.0 (11.8–88.2) |
| Stable disease | 6 | 31.6 (12.6–56.6) | 2 | 33.3 (4.3–77.7) |
| Progressive disease | 1 | 5.3 (0.1–26.0) | 0 | 0.0 (0.0–45.9) |
| Nonevaluable | 1 | 5.3 (0.1–26.0) | 0 | 0.0 (0.0–45.9) |

5-FU, 5-fluorouracil; CI, confidence interval.

+ indicates that there was no progressive disease at last disease assessment.

^a^Confirmed by repeat radiographic assessment ≥4 weeks after first documentation of response.

^b^Based on binomial exact CI method.

^c^Complete response + partial response.

^d^Complete response + partial response + stable disease maintained for ≥6 months.

**Figure S1.** CONSORT diagram (**a**) cohort 2 and (**b**) cohort 3.

**
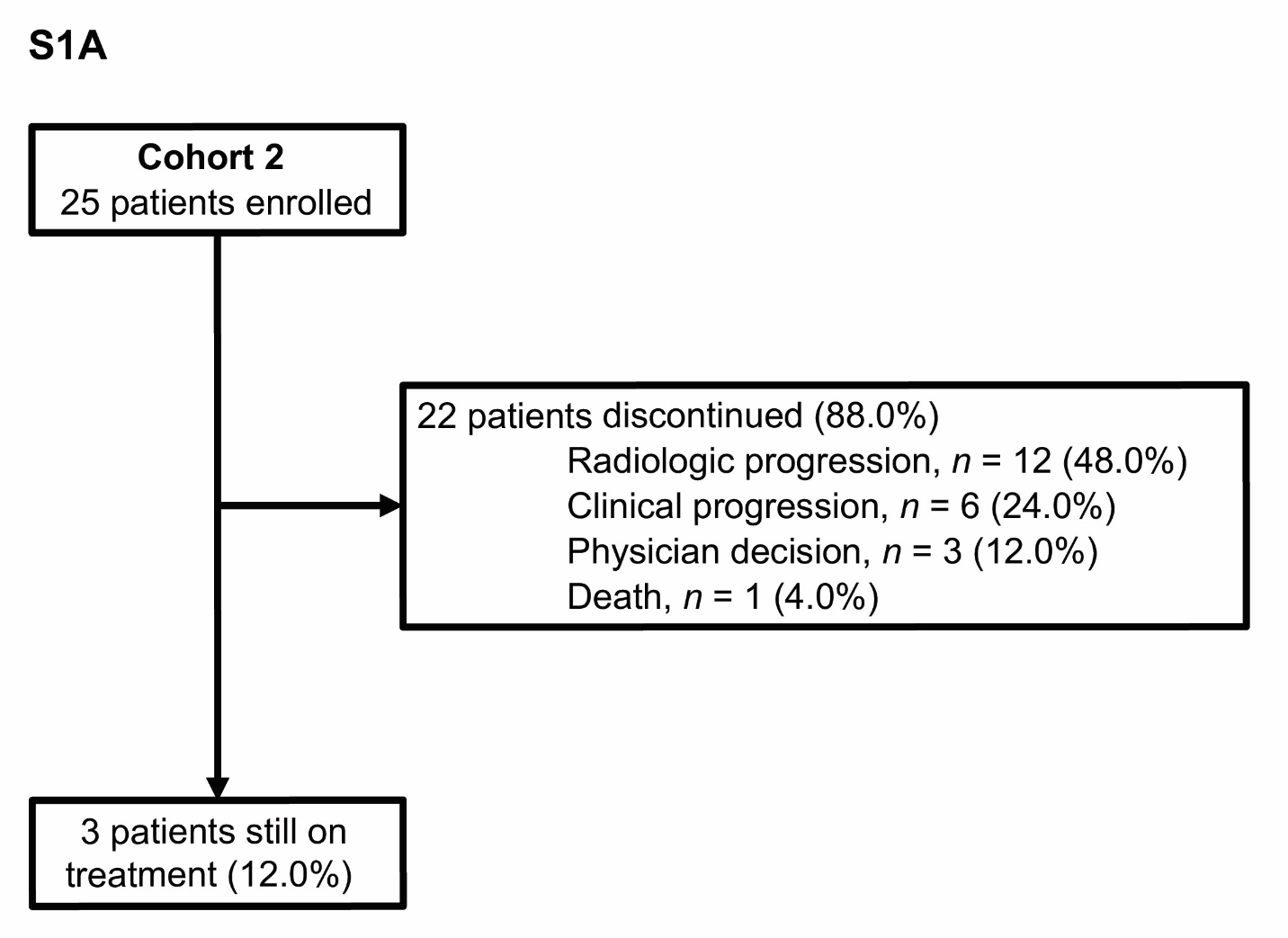
**

**
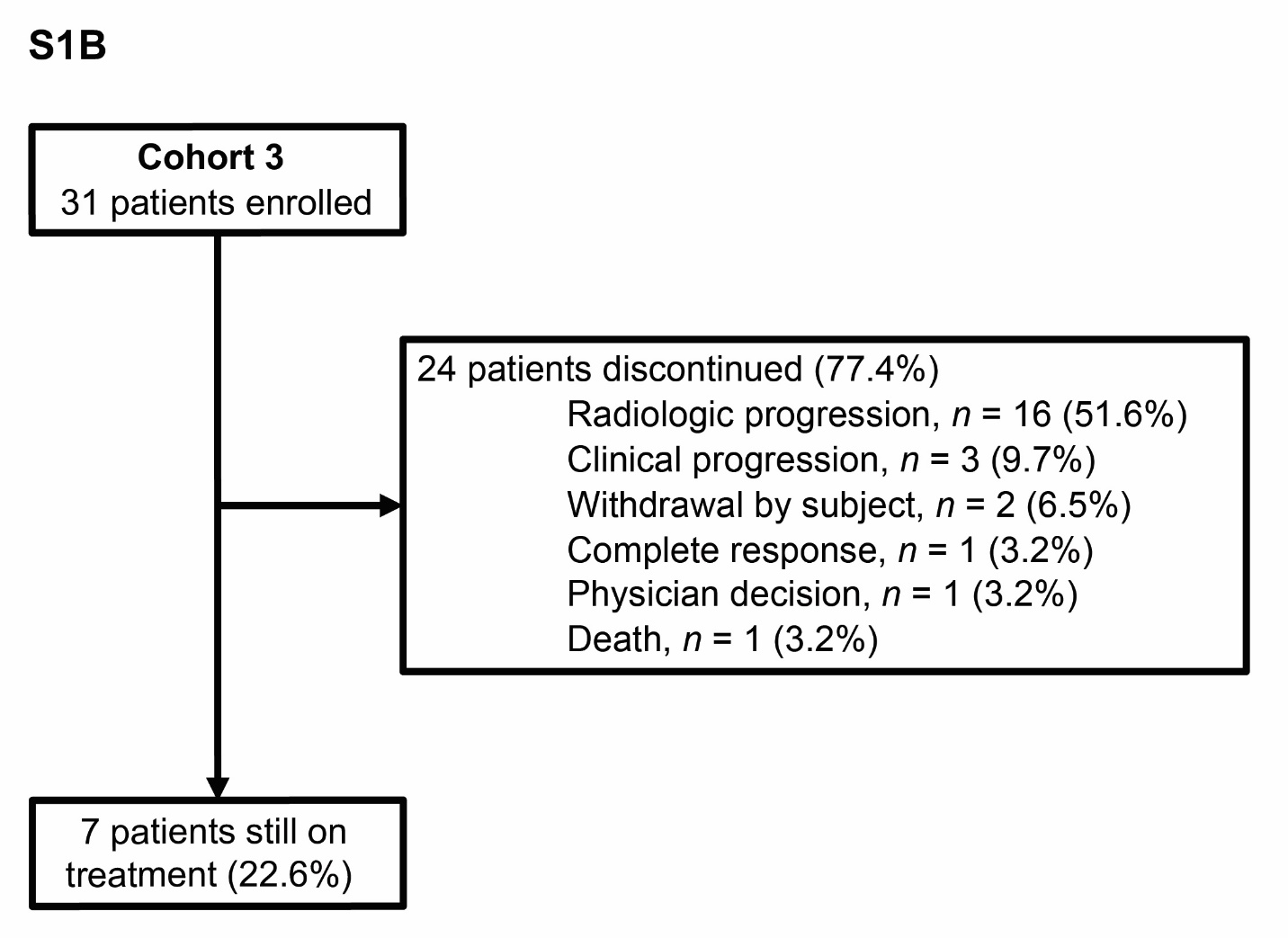
**

**Figure S2.** Kaplan-Meier estimates of duration of response among confirmed responders in (**a**) cohort 2 (*n* = 15) and (**b**) cohort 3 (*n* = 8).

**
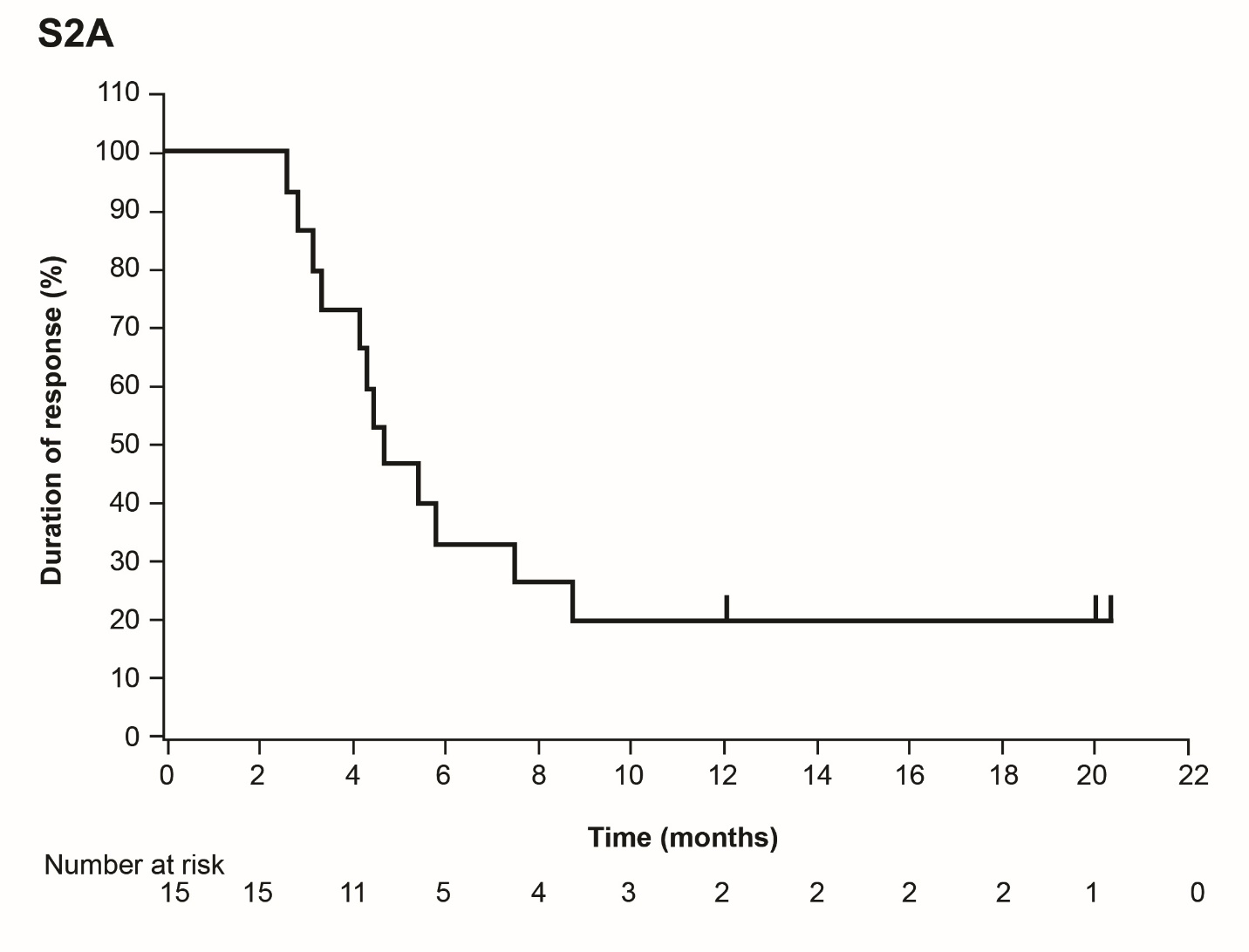
**

**
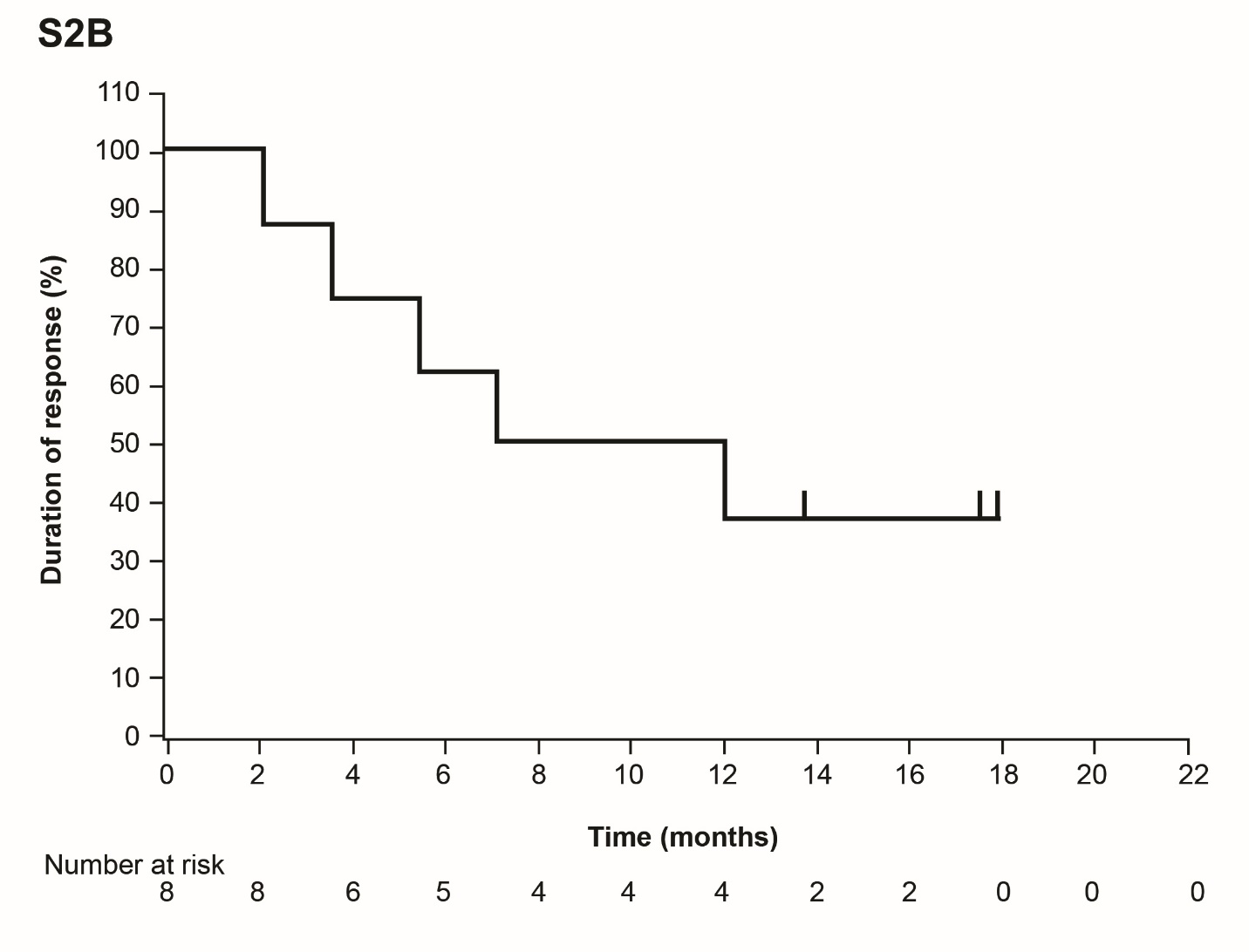
**

**Figure S3.** Longitudinal change from baseline in the sum of the longest target lesion diameters per patient in (**a**) cohort 2 (*n* = 24)^a^ and (**b**) cohort 3 (*n* = 28).^a^
^a^Patients with measurable disease per RECIST v1.1 by central review at baseline who had ≥1 evaluable postbaseline assessment. RECIST, Response Evaluation Criteria in Solid Tumors.


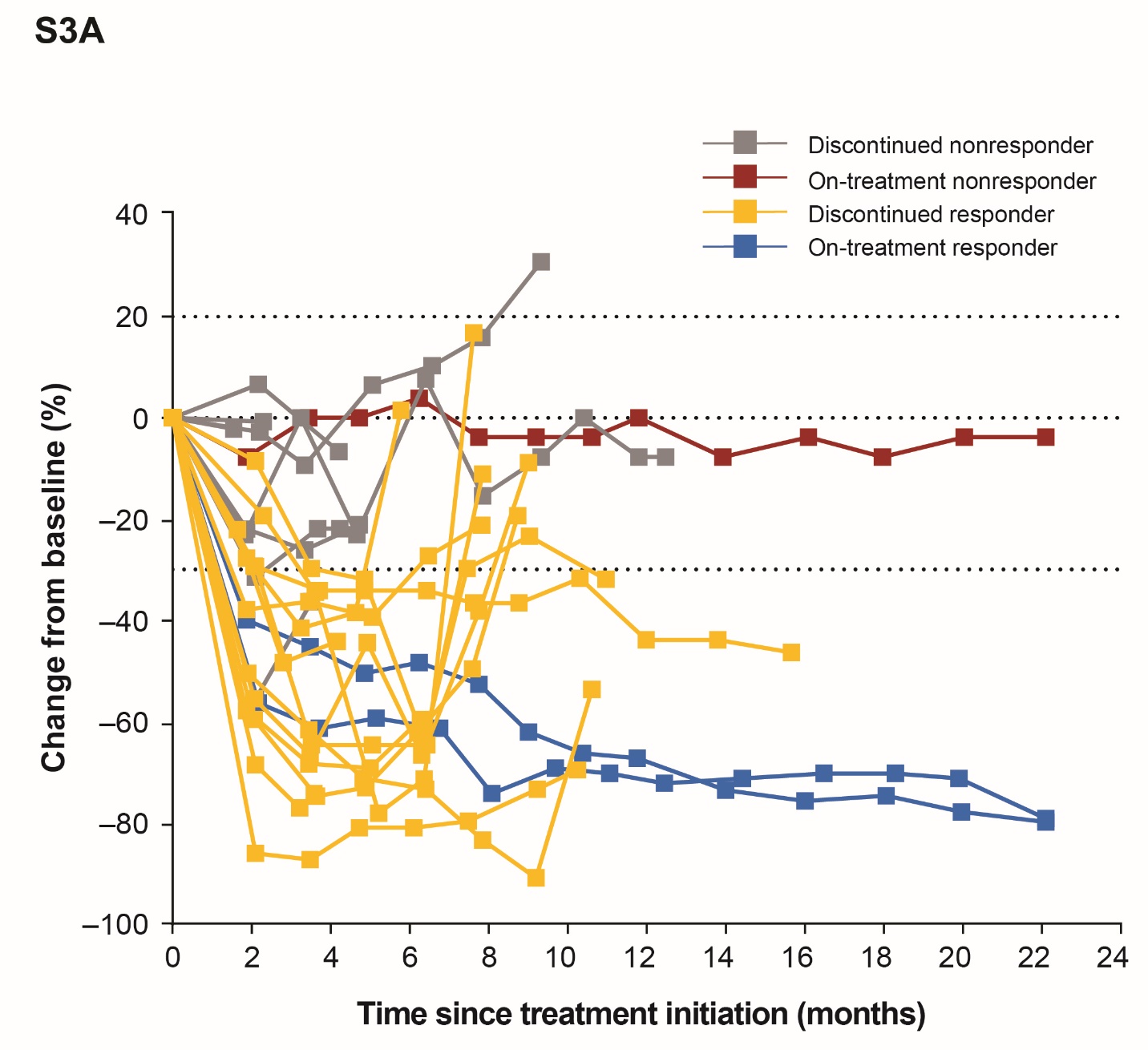


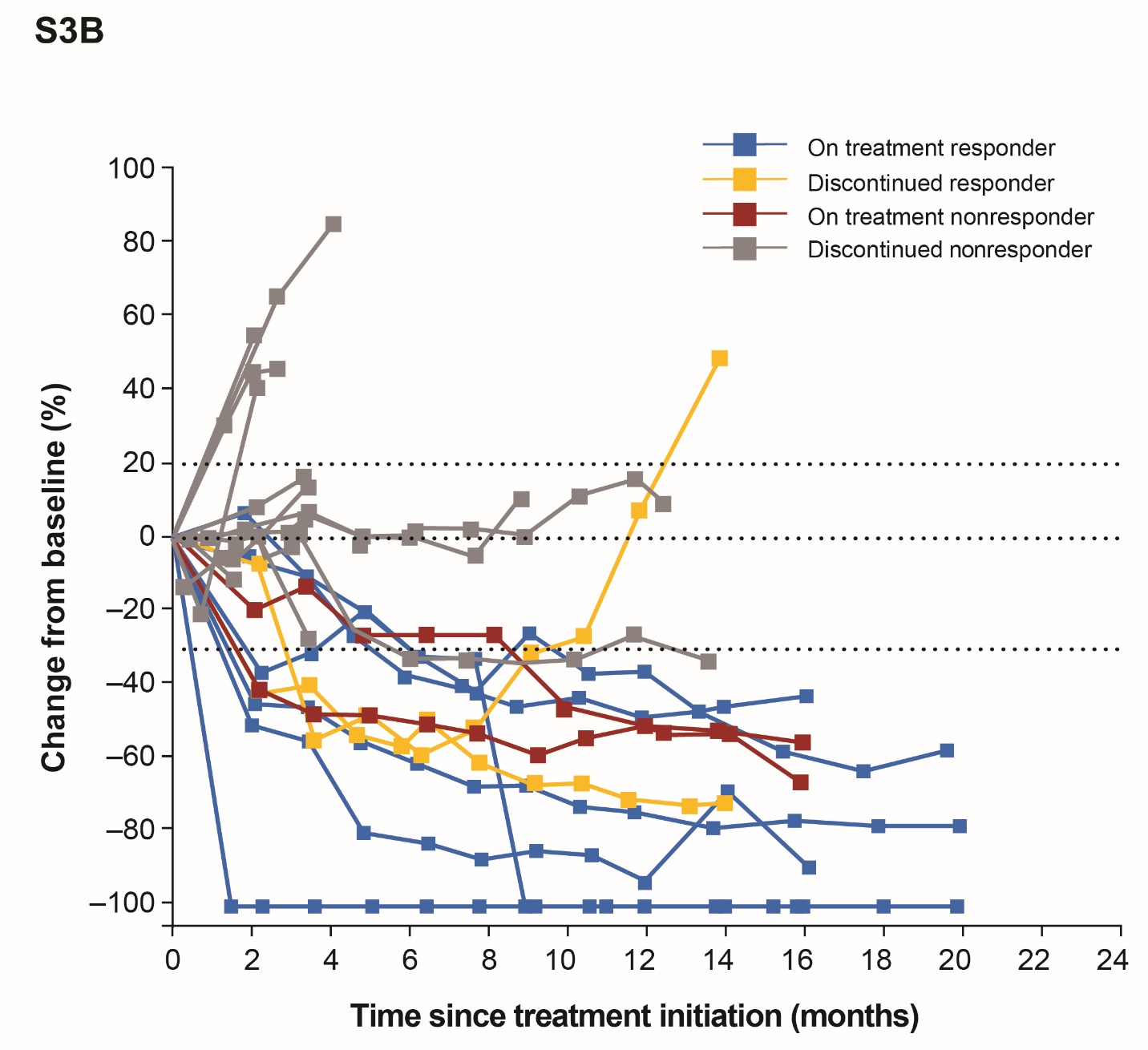

Supplement: Supplementary file 1 — Supplementary material 1 (DOCX 1069 KB) [file 10120_2018_909_MOESM1_ESM.docx]
